# TRAK MSK: a randomised external feasibility trial and process evaluation of a digital physiotherapy supported self-management programme for people with musculoskeletal pain

**DOI:** 10.1186/s12891-026-10170-y

**Published:** 2026-07-04

**Authors:** Button Kate, Dean-Young Andrew, Anderson Pippa, Cullen Katherine, Jacob Nina, Jones Fiona, Letchford Robert, Meister Lena, Pellow Andrew, Playle Rebecca, Randell Elizabeth, Rice John, Busse-Morris Monica

**Affiliations:** 1 Cardiff University, Heath Park, Cardiff, Wales CF14 4XN United Kingdom; 2 Sirona Care and Health, Bristol, BS15 9TR United Kingdom; 3 Cardiff and Vale University Health Board, Heath Park, Cardiff, CF14 4XW United Kingdom; 4 Swansea University, Singleton Campus, Swansea, SA2 8PP United Kingdom; 5 City St Georges, University of London GB, London, EC1V 0HB United Kingdom; 6 Powys Teaching Health Board, Mansion House Bronllys, Brecon, LD3 0LY United Kingdom; 7 Cwm Taff Morgannwg University Health Board, Royal Glamorgan Hospital, Ynysmaerdy, CF72 8XR United Kingdom; 8 Florence Nightingale Faculty of Nursing, Midwifery and Palliative Care, Kings College London, London, SW1Y 4AN United Kingdom

**Keywords:** Digital, Musculoskeletal, Physiotherapy, Self-management support

## Abstract

**Background:**

TRAK MSK is a co-designed digital physiotherapy supported self-management programme. It enables people to develop the knowledge, confidence and skills to self-manage their condition and pain, to improve everyday function and physical activity levels. We explored the feasibility of conducting a definitive trial of the TRAK MSK intervention.

**Methods:**

We conducted a two-arm multi-site randomised external feasibility trial with an embedded process evaluation. We recruited adults aged 18 and over with self-reported activity related joint pain for at least 3 months, attending physiotherapy clinics. Participants were randomised to either the TRAK MSK intervention or usual NHS physiotherapy care. The TRAK MSK intervention was delivered over 16 weeks and included up to five online coaching consultations with a physiotherapist trained in self-management support, and access to a website designed to support these consultations. Primary feasibility outcomes were recruitment, randomisation and retention at 16 weeks post-randomisation, all of which were pre-defined. Secondary outcomes were feasibility of collecting health services resource use and intervention costs and reported variability of participant rated questionnaires at baseline and follow up. We evaluated acceptability of intervention receipt and trial processes and intervention fidelity with an embedded process evaluation.

**Results:**

We consented 87 (59%) eligible participants from 147 that expressed an interest in taking part. Participants needed to complete baseline outcome measures to progress to randomisation. Seventy-five individuals (86% of those consented and 51% of those who expressed interest), were randomised and received TRAK MSK intervention (*n* = 37) or usual care (*n* = 38); 12 consented participants did not complete their baseline outcome measures and were not randomised. After 16 weeks, 23 participants from TRAK MSK and 22 participants from usual care completed the participant rated questionnaires (60%). Interviews with participants and physiotherapists suggested the intervention was acceptable and had a place in future ways of working. Outcomes reflecting self-rated pain, physical activity, confidence in managing pain and health related quality of life were in the anticipated direction and aligned to the TRAK MSK programme theory.

**Conclusion:**

Progression to a full trial is recommended based on feasibility and acceptability with modifications, such as using multiple recruitment strategies and initial in person visit. The definitive trial design will be informed by the findings of this study and stakeholder engagement. Physiotherapist training for self-management support should include components that build the skills to integrate patient digital health literacy coaching into TRAK MSK.

**Trial registration:**

ISRCTN82011326. Registration date: 15/07/2022. Date of first participant enrolment 01/07/2022. This trial was retrospectively registered.

**Supplementary Information:**

The online version contains supplementary material available at https://doi.org/10.1186/s12891-026-10170-y.

## Background

Musculoskeletal pain is associated with loss of function and independence during everyday activities, lower levels of physical activity and reduced overall well-being [1, 2]. Globally approximately 1.71 billion people live with musculoskeletal conditions [3]. With an aging population this number is set to increase, placing greater burden on health resources [1]. Therefore, new approaches are needed that enable people to care for themselves outside of the hospital and allow them to be more involved in making decisions about their care and health [4].

Support for self-management is considered a core treatment for chronic musculoskeletal pain. It enables individuals to develop the skills and confidence to manage their condition, with less reliance on health services and be more active, with higher levels of function during everyday activities [5]. However, physiotherapists often lack the time, experience, skills, confidence and training to integrate personalised self-management support and coaching techniques, so further help is needed [6, 7].

Digital interventions that include routine and innovative forms of information and communications have the potential to improve the quality and sustainability of how physiotherapy for musculoskeletal pain is delivered, with care accessible from home [8–10]. However, few digital self-management and exercise interventions have been developed using formalised approaches to intervention development and testing and underpinning programme theory [11–16].

TRAK musculoskeletal (TRAK MSK) has been developed in an iterative process using theory and evidence-based approaches as well as person centred approaches [7, 17–23]. The Medical Research Council framework for developing and evaluating complex intervention has guided the development [24]. Delivered by physiotherapists TRAK MSK digital self-management support intervention allows treatment to be delivered in the patient’s own home and help them remain physically active. In TRAK MSK an online consultation conducted with a physiotherapist trained in self-management support is combined [25, 26] with access to the TRAK MSK website, which has been optimised to support self-management.

**Fig. 1:**
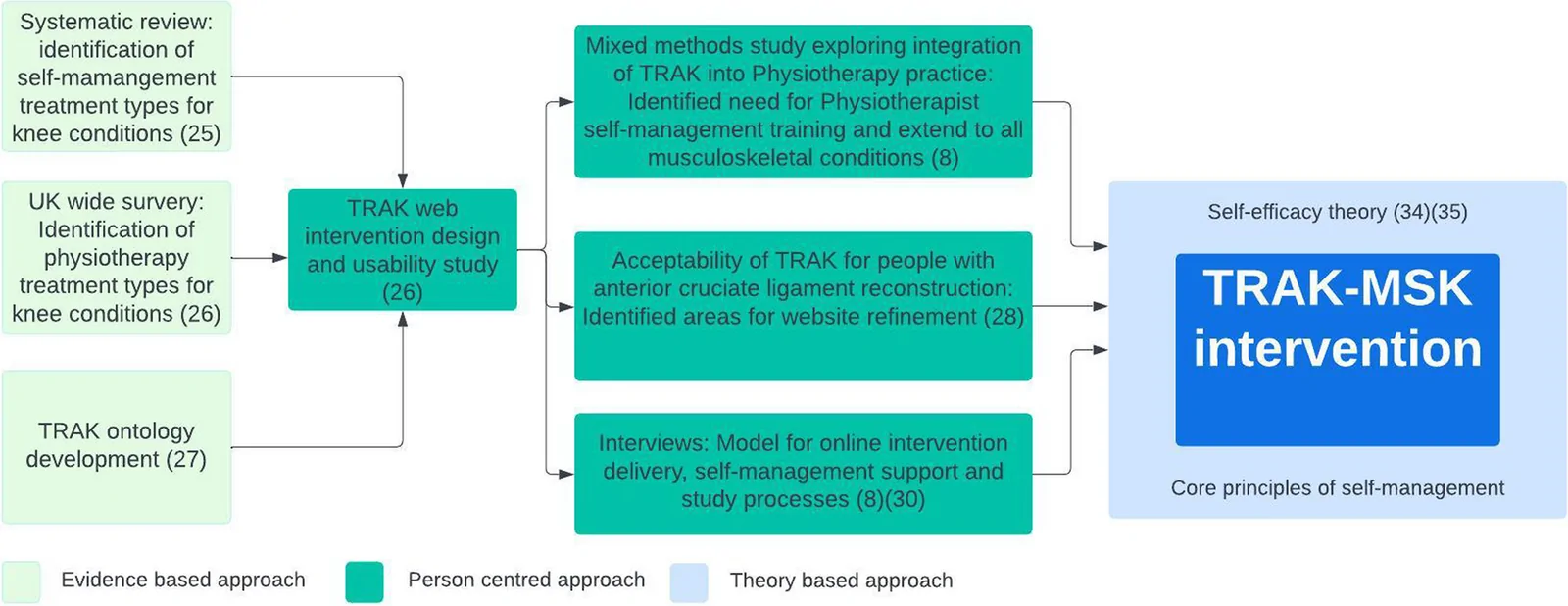
Development of TRAK MSK

The developmental approaches and process are detailed in Fig. 1. The initial TRAK offering was designed to be a self-treat website based on the integration of evidence from a series of studies [25–27]. However, user feedback and evaluations revealed that the website was most effective when used alongside physiotherapy support for self-management. This combined approach improved both engagement and potential therapeutic benefit [26].

Further person-centred studies identified the importance of supporting patients in building knowledge, confidence and skills to make informed decisions about their health [8]. It also identified the need for tailored training for physiotherapists to help them facilitate a self-management approach [8].

To address these needs, the intervention incorporated specific sources of self-efficacy such as personal goal setting, self-discovery and mastery of experiences (Table 1), based on stakeholder input [8]. The benefits of enhanced self-efficacy in improving quality of life have been demonstrated in other conditions [28, 29].

**Table 1:** Core principles of self-management

Core Principles	Definitions	Language Examples
Personalised goals	What matters most to the person, focussing on small steps in the here and now as well as acknowledging future hopes	What is one small thing you want to do right now? What would help you feel like you?
Problem-Solving	Supporting people to think through problems and come up with strategies together	What has worked for you before?
Reflection on mastery experiences	Supporting people to reflect on what’s working, to recognise and record their strengths and capabilities	How does that compare to last week?
Personal communities	Supporting people to access their support network (family, friends, community) as well as community resources	Who do you know that could help?
Self-Discovery	Supporting people to try new things, or approach old tasks in new ways. May involve risk-taking	What does it feel like to you?
Knowledge of self and condition	Supporting people to develop greater self-awareness and giving people meaningful information	What do you understand about…?
Taking Action	Supporting people to do more in their everyday lives, even small things, and appraising their efforts	What is stopping you giving it a go?

The TRAK MSK intervention was subsequently expanded to support individuals with musculoskeletal pain across all joints. This extension was informed by feedback from physiotherapists, patients, and public contributors, aiming to reduce implementation barriers and address the complexity of co-existing musculoskeletal conditions. TRAK MSK fosters a collaborative environment where physiotherapists and participants work in partnership. This approach empowers individuals to make decisions about their health and through improved self-efficacy, gain greater confidence in managing their musculoskeletal condition and daily activities [8, 30, 31].

The aim of this study was to evaluate the feasibility of conducting a future pragmatic, multi-centre randomised controlled trial, to compare the TRAK MSK digital physiotherapy self-management intervention to usual care. This aligns with the next phase of the MRC guidance on testing and developing complex interventions [24]. The primary feasibility objectives were to evaluate the number of participants recruited per month per site, willingness of participants to be randomised and completeness of patient rated outcome measures (PROMs) at 16 weeks post-randomisation. All objectives were pre-defined. Secondary objectives included assessing the feasibility of collecting data on health resource use and intervention costs to inform future evaluations, measuring the variability of PROM scores at baseline and follow up and evaluating the fidelity of intervention delivery. The acceptability of the intervention and trial processes were explored through an embedded process evaluation [32].

## Methods

### Study design

We conducted a two-arm individually randomised external feasibility trial [33] comparing the TRAK MSK intervention to usual care, with an embedded process evaluation. This definition of feasibility follows the Consort statement extension for randomised pilot and feasibility trials, to which this study adheres [31, 32].

### Participants and recruitment

We aimed to recruit 80 participants aged over 18 and who had pain affecting a joint (with pain originating from the back, legs or arms) for more than 3 months with pain on most days of the past month, which worsened with activity [6], and who had been referred or self-referred to physiotherapy. Participants were required to have capacity to consent. Individuals were excluded if exercise was contraindicated, if their pain was caused by malignancy, fractures or inflammatory arthritis or they had received surgery for their musculoskeletal pain in the last 12 months. Where possible, participants were requested to have access to a mobile or internet connection at home, with options to loan a device or have telephone support if they did not have access. The sample size was based on a 95% confidence interval width of no more than plus or minus 10 percentage points attainable using a point estimate of 70% for retention. This exceeds the recommendations of Teare et al. 2014 [33], who used a simulation approach to calculate the sampling distribution for the standard deviation of continuous outcomes.

We recruited across three Health Boards (Site A, B and C) in Wales either by self-referral or referral from clinical waiting lists. The sites were based in out-patient physiotherapy services, spread across Mid and South Wales and varied in terms of population density, social-economic deprivation and ethnic groups. For self-referral the study was advertised on social media or on the website of participating sites. For those recruited via clinical waiting lists, all new referrals were screened by a Physiotherapist, and potential participants were given a patient information sheet in clinic or sent an email advertising the study. All interested participants could then enrol in the study using the TRAK MSK website (www.myjointpain.org.uk). Participants were required to complete an online eligibility check and consent form, followed by online baseline questionnaires. If a participant did not have access to the internet they could contact a member of the research team by telephone.

### Randomisation

Following completion of baseline outcome measures participants were randomised to treatment or control arm (1:1 ratio) with a 2:1:1 ratio for strata (site). The largest site was expected to recruit twice as many as the other two sites. Random permuted blocks stratified by site were used to ensure similar numbers in the treatment groups throughout the trial. This was done electronically by the data manager who did not have any direct contact with participants, and all baseline questionnaires were completed independently by participants at home. The sites were advised on the group allocated to and a physiotherapist appointment was then arranged.

### Intervention

The intervention had three components (1) Physiotherapists trained to deliver self-management support using a coaching approach, (2) participant access to TRAK MSK website with interactive functionality to support self-management from home (3) online consultations.

All physiotherapists delivering the intervention received training in self-management support. The goal was to equip TRAK MSK practitioners with the knowledge, skills and confidence needed to support individuals with joint pain in managing their condition. The training consisted of three interactive, two-hour online sessions, based on the Bridges Self-Management approach. This approach has been successfully developed and evaluated in stroke, brain injury and other long-term conditions [28, 29, 34, 35].

Delivered by Bridges facilitators, the training focused on building skills for personalised coaching, guided by goals for activities that are meaningful to each individual. The Bridges approach is grounded in the concept of self-efficacy and incorporates seven core self-management principles [36, 37] (Table 1).

The training was tailored to the experiences, understanding, and needs of physiotherapists working with people who have joint pain. It included interactive activities, films, role play, prompts, and guides to help practitioners reduce the amount of information and direction they give and instead support the development of shared self-management plans from the very first consultation.

The functionality of the TRAK MSK website (www.myjointpain.org.uk) could only be accessed via a unique website login. The website was developed to complement a self-management approach with a goal-setting function, an activity suite for patient participants to develop personalised activity/exercise plans and access to email support from the physiotherapist. The website also contained information on pain, exercise and healthy behaviours that was accessible to the intervention and control group. The website design was responsive, making it easily accessible on any device and accessible from the clinic and the participant’s home. Participants were given full access to the platform for 6 months. Physiotherapists could also view the goals and exercise playlists of participants they were treating.

All one-to-one coaching sessions were conducted online through the platform adopted in each Health Board. Participants were offered up to 5 online coaching consultations over 16 weeks. The number received was based on shared decision making between physiotherapists and participants. Physiotherapists were required to complete a practitioner questionnaire following each consultation to capture information on self-management concepts used.

### Comparator

The comparator was usual care physiotherapy. This could be an online consultation using the platform used at site, or face-to- face physiotherapy, with a physiotherapist that had not received the coaching training in self-management support. Usual care participants could access information on pain, exercise and healthy behaviours on the TRAK MSK website but did not have access to any other website functionality, which could only be accessed via a login by the intervention participants. There was no limitation on the number or the timeframe of usual care consultations.

### Outcomes

The primary outcomes for the external feasibility trial were:

- Recruitment rate (the proportion of eligible participants recruited and randomised for each site per month).
- Willingness to be randomised after consenting (randomisation rates %)
- Follow-up rates (proportions completing 16-week follow-up assessment of PROMs).

The secondary outcomes were:

- Safety and adverse events reported by the treating physiotherapist.
- PROMs at baseline and 16 weeks follow-up, collected online via the TRAK-MSK website. We assessed means and standard deviations for; Musculoskeletal Health Questionnaire with higher scores (total possible range 0–56) indicating better musculoskeletal-HQ health status (MSK-HQ), Musculoskeletal Physical Activity sub-scale (MSK-PA) with higher scores indicating higher levels of physical activity (range 0–7) [38], Numeric Rating Scale with lower scores (total possible range 0–10) reflecting less pain [38], Pain self-efficacy scale with higher scores (total possible range 0–60) reflecting stronger self-efficacy beliefs [39], Patient Health Questionnaire for Depression where lower scores (total possible range 0–27) indicate less depression (PHQ-9) [40], General Anxiety Disorder Scale with lower scores (total possible range 0–21) indicating less anxiety (GAD-7) [41]. EQ-5D 5L and EQ5D visual analogue scale (EQ5D-VAS) [29] were a measure of health state (higher scores indicating a better health state) and the ICEpop Capability Measure for Adults ICECAP-A [42] a measure of capability. EQ-5D 5L and ICECAP-A were collected to explore their potential for use in economic evaluation.
- Participant-rated health and care service resource use related to implementation of the TRAK MSK intervention or usual care patient pathways and related patient outcomes was collected, to understand major resource drivers to inform future study planning. A self-reported resource use measure (RUM) was developed with input from the research team, including the patient and public involvement (PPI) group (supplementary file 1). The viability of data collection was assessed to determine whether the measures could be completed by participants.

### Analysis

All demographic data for baseline measures were summarised as frequencies and percentages, means (SD) or median [interquartile range] as appropriate to the distribution of the data, using SPSS v27 and STATA v17. PROMs at baseline and 16-week follow up were scored according to published algorithms or rubrics and presented descriptively. An intention to treat approach was used. The analysis team were blinded to which group participants were randomised. There was no imputation for missing data as per guidelines for external feasibility trials [32] The mean cost per participant of NHS health and social care were presented by study arm at baseline and 16 weeks, as well as the personal costs at these time points. Unit costs were derived from published sources [43–46] and EQ5D-5L data were mapped to the 3L valuation set [47].

Assessment of pre-specified progression criteria was based on a traffic light system (green/amber/red) [48], with green meaning progress to full trial, amber meaning progress with modifications so that the criteria would be achieved in a full trial and red meaning stop with no progression to a full trial (see Table 2).

**Table 2:** Progression criteria and traffic light assessment

Primary Outcomes			
Progression Criteria	Traffic Light Assessment		
	Green	Amber	Red
Recruitment rate: Recruited participants per month per site (A, B or C)	10/5/5	8/3/3	6/2/2
Willingness to be randomised	70% or higher	50–70%	49% 0r lower
Completeness of PROMS outcome data at 16 weeks post- randomisation [8, 49]	70% or higher	60–70%	59% or lower

### Process evaluation

Key outcomes that were evaluated as part of the process evaluation were:

Acceptability of the intervention through patient and physiotherapy interviews on confidence to self-manage, digital literacy, technology acceptance, experience of the intervention, trial processes and barriers and facilitators to participating. All Interviews were carried out online by a member of the research team (ADY) and audio recorded using Microsoft Teams. An interview schedule was created based on our previous work [8] (supplementary file 2). The audio files were transcribed using a transcription service. Interviews were coded inductively using thematic analysis by a member of the research team (NJ) once all the interviews were completed [47]. All physiotherapists that delivered the intervention were invited to an interview. All patient participants that completed the TRAK MSK intervention and 16-week follow-up were invited to an interview using convenience sampling.

Fidelity of delivery of TRAK MSK by healthcare professionals was evaluated through self-report on a digital form. This incorporated the core principles used in each consultation with examples. This was reviewed by a member of the trial team and summarised descriptively. Adherence with the intervention based on the number of online consultations attended. All participants were expected to attend one session.

A process evaluation framework was used to help us consider wider contextual factors related to the feasibility trial design and delivery [31]. With the interviews occurring post-intervention it provided an opportunity to explore experiences of participating in the trial as well as views on the treatment provided and the wider contextual issues related to the intervention and its accessibility e.g., digital inclusivity and uptake in underserved groups.

### Trial Steering Committee

The Trial Steering Committee was made up of an independent statistician, health economist and experienced rehabilitation researcher. The chief investigator and trial statistician also attended. The committee met 3 times over the course of the study to have overview and give guidance on procedures, progress and future recommendations. The results and achievement of progression criteria were reviewed by the committee.

## Results

We opened three sites to recruitment. Sites were actively recruiting between November 2022 to end of March 2023. Site A recruited 40 participants; Site B recruited 33 participants and Site C recruited 14 participants. This equated to a recruitment rate per month per site of 10, 8 and 5 respectively (green traffic light assessment).

Across all sites, we received 147 expressions of interest. Of these, 87 (59%) individuals consented to take part but 12 of these failed to complete their baseline questionnaires, so 75 (86%) were randomised, with 37 participants allocated to the intervention arm and 38 to usual care. This equated to a green traffic light assessment for willingness to be randomised. Post randomisation one participant from the intervention arm was inadvertently booked for a usual care appointment due to an administration error so they continued in the usual care arm (but were analysed using intention to treat). Training was provided to make sure this did not happen again.

Twenty-three participants in the TRAK MSK intervention arm and 22 in the usual care arm completed the requested PROMs at follow up equating to a 60% completeness of outcome data at 16 weeks post- randomisation (amber traffic light assessment). All follow ups were completed by end of June 2023. Findings for the progression criteria are given in Table 3. Following the traffic light assessment, we established before commencing the trial we achieved amber or green for each criterion.

**Table 3:** Participant demographic characteristics

	Intervention mean (*N* = 36)*	Control mean (*N* = 37)*
Age (years)	56 (12)	53 (15)
	Intervention *N* = 36 (%)	Control *N* = 37 (%)
Gender identify as (*N* = woman)	22 (61.1)	26 (70.3)
Sex at Birth (*N* = female)	22 (61.1)	26 (70.3)
Ethnicity		
Welsh/English/Scottish/Northern Irish/British	34 (94.4)	35 (94.6)
Caribbean	0 (0)	1 (2.7)
White and black Caribbean	0 (0)	1 (2.7)
Other white background	1 (2.8)	0 (0)
Other mixed/multiple ethnic background	1 (2.8)	0 (0)
Living:		
Alone	3 (8.3)	5 (13.5)
With partner	17 (47.2)	15 (40.5)
With children	2 (5.6)	4 (10.8)
With Partner & children	13 (36.1)	11 (29.7)
With other family members	0 (0)	2 (5.4)
With other non-family	1 (2.8)	0 (0)
Highest qualification		
None	2 (5.6)	2 (5.4)
1–4 GCSEs^$^ or equivalent	1 (2.8)	4 (10.8)
5 + GCSE or equivalent	2 (5.6)	7 (18.9)
Apprenticeship	0 (0)	1 (2.7)
A levels or equivalent	7 (19.4)	4 (10.8)
Degree or above	22 (61.1)	19 (51.4)
Vocational qualifications	2 (5.6)	0 (0)
Employment status		
Full time education	4 (11.1)	2(5.4)
Part time education	0 (0)	1 (2.7)
House person	1 (2.8)	0 (0)
Employed full time	15 (41.7)	17 (45.9)
Employed part time	3 (8.3)	4 (10.8)
Unemployed	3 (8.3)	3 (8.1)
Retired	10 (27.8)	10 (27.0)
Current job	*N* = 18 (%)	*N* = 21 (%)
Corporate managers and directors	2 (11.1)	2 (9.5)
Science, research, engineering & technology professionals	1 (5.6)	2 (9.5)
Health professionals	1 (5.6)	1 (4.8)
Teaching and educational professionals	2 (11.1)	3 (14.3)
Other managers and proprietors	1 (5.6)	1 (4.8)
Health and social care associate professionals	1 (5.6)	1 (4.8)
Culture, media and sports occupations	0 (0)	1 (4.8)
Skilled metal, electrical and electronic trades	1 (5.6)	2 (9.5)
Skilled construction and building trades	0 (0)	1 (4.8)
Administrative occupations	5 (27.8)	2 (9.5)
Secretarial and related occupations	0 (0)	1 (4.8)
Caring personal service occupations	0 (0)	1 (4.8)
Customer service occupations	0 (0)	1 (4.8)
Other Job not listed	4 (22.2)	2 (9.5)
Joint area	*N* = 31 (%)	*N* = 36(%)
Spine	5 (16.1)	7 (19.4)
Upper limb	10 (32.2)	15 (41.6)
Lower limb	16 (51.6)	14 (38.8)

^*^Post randomisation one participant was offered and took up usual care. This was recorded as a protocol deviation, but the participant remained in the trial and data were analysed using intention to treat

^$^General Certificate of Secondary Education (GCSE)

**Fig. 2:**
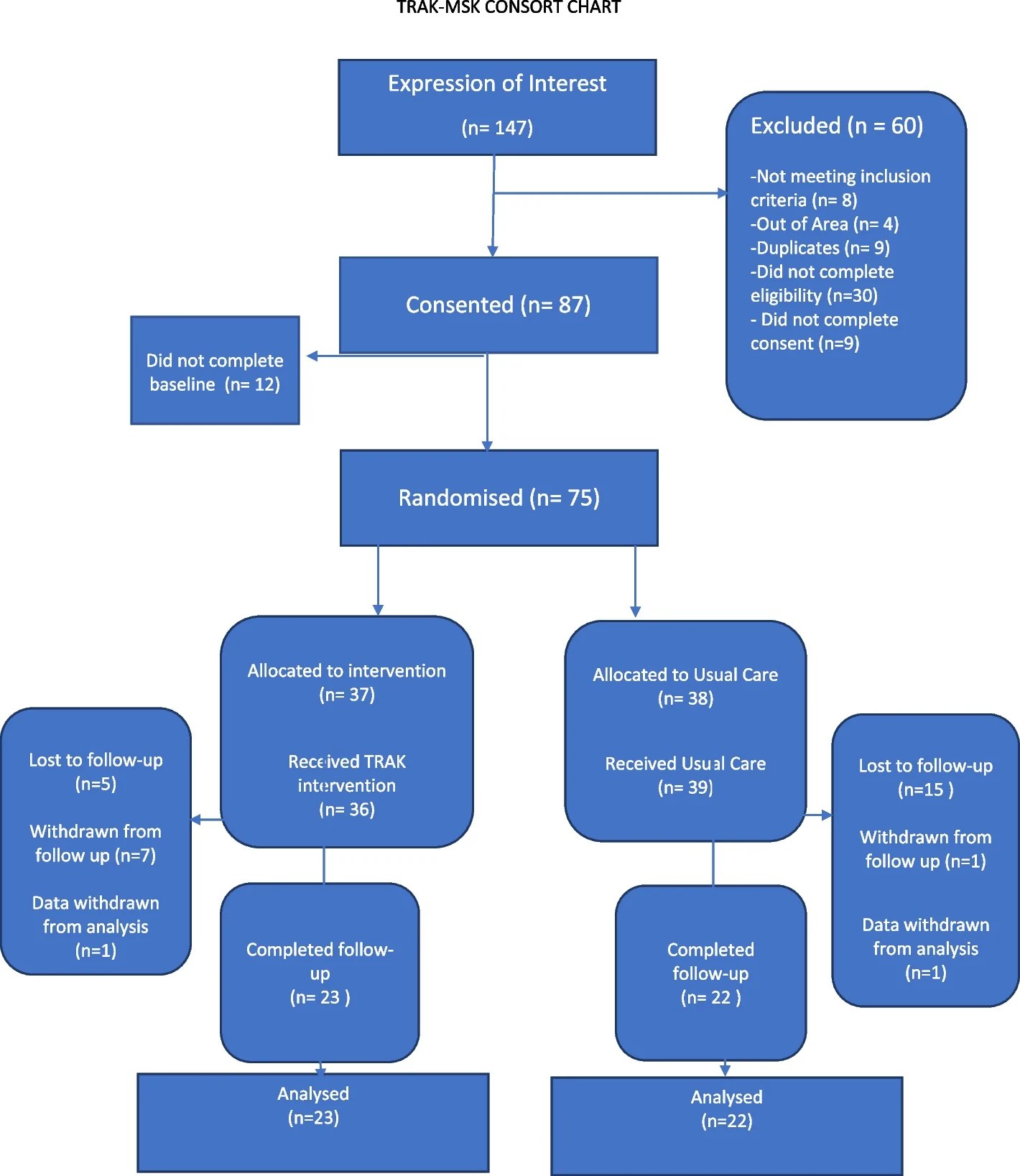
Consort flow chart of participants

The consort diagram outlining the flow of participants through the study is illustrated in Fig. 2.

### Participant characteristics

The characteristics of the participants that took part in the study are presented in Table 3. Based on descriptive data both groups were similar at baseline.

### Safety

No safety issues were identified throughout the trial.

### Patient reported outcome measures

Mean and Standard Deviation (SD) scores for the PROMs at baseline and at 16 week follow up for the intervention and control group are presented in Table 4. Scores on MSK-HQ, MSK-PA, EQ-5D and pain self-efficacy score were higher in the TRAK intervention group. Numeric pain scores were lower. Scores on EQ5D-VAS, GAD7 and PHQ9 were marginally indicative of worse health on the day and greater anxiety and depression at follow up. There were also improvements in the MSK-HQ in those who received usual care but minimal changes in other PROMs at follow up.

**Table 4:** Patient reported outcome measures

	Intervention (*N* = 23)		Usual Care (*N* = 22)	
	Baseline Mean (SD)	Follow-up Mean (SD)	Baseline Mean (SD)	Follow-up Mean (SD)
Musculoskeletal Health Questionnaire (MSK-HQ)	36.7 (8.1)	43.1 (8.4)	29.6 (8.7)	36.5 (11.4)
Musculoskeletal Physical Activity (MSK-PA)	3.3 (2.2)	4.0 (1.8)	3.3 (2.4)	3.4 (2.0)
Pain Numeric Rating Scale (NRS)	5.4 (1.9)	3.2 (2.2)	5.6 (1.7)	4.3 (2.3)
General Anxiety Disorder assessment (GAD7)	2.7 (2.3)	3.2 (2.6)	5.2 (3.8)	5.5 (5.3)
Patient Health Questionnaire (PHQ9)	4.0 (2.9)	4.3 (4.1)	5.8 (5.0)	5.7 (5.6)
Euroqol 3 dimensions (EQ5D-5L)	0.66 (0.2)	0.72 (0.1)	0.61 (0.1)	0.60 (0.1)
Euroqol Visual Analogue Scale (EQ5D VAS)	78.3 (15.5)	73.7 (20.8)	70.8 (13.2)	72.1 (19.7)
Pain Self-Efficacy Questionnaire (PSEQ)	45.4 (10.4)	50.3 (11.8)	41.2 (10.3)	43.1 (15.3)
ICEpop CAPability measure for Adults (ICECAP-A)	0.90 (0.10)	0.88 (0.13)	0.86 (0.16)	0.83 (0.14)

### Healthcare resource use

Healthcare resource use was well reported with few missing data points. However, there were several items that were not reported to be used by any participant and could be removed from the final RUM, such as hospital admissions and impact of COVID-19. A full report of healthcare resource use is given in supplementary file 3.

### Intervention costs

Records of TRAK MSK sessions were reported for 28 participants. Only 4 participants (5.1%) had the maximum 5 sessions. The duration of the sessions was from 5 to 60 min (mean = 30, SD 13), applying the mean cost of a band 5/6 physiotherapist time (early and specialist) [50] gave staff costs per session ranging from £4 to £46.80 (mean cost = £23.69, SD 10.3). The total cost of physiotherapy time for TRAK MSK sessions was £1,682 for the sessions reported. These costs do not include time for administration, note-taking, or training.

At 16 weeks the number of people who had physiotherapy appointments in the community or primary care were similar (TRAK *N* = 3, Usual care *N* = 4), however, there were more people having physiotherapy outpatient hospital appointments in the usual care arm (TRAK *N* = 1, Usual care *N* = 9). Applying NHS reference costs to community physiotherapy appointments (£73.14) and outpatient appointments (£100) [50] resulted in higher costs for physiotherapy in the usual care arm (Table 5).

**Table 5:** Physiotherapy appointment costs for all TRAK MSK and usual care arm participants at baseline and16-week follow-up

Physiotherapy appointments	Baseline		16 weeks	
	TRAK (*N* = 36) N people, cost	Usual care (*N* = 37) N people, cost	TRAK (*N* = 20) N people, cost	Usual care (*N* = 22) N people, cost
NHS -Community	4, £365.67	7, £511.98	3, £585.12	4, £804.54
NHS—Hospital	2, £200	1, £100	1, £100	9, £2,300
Private*	3, £220	2, £152	0	0
NHS physiotherapy costs	£ 565.67	£611.98	£685.12	£3,104.54

^*^mean cost reported—£47 per session

### Process evaluation

#### Physiotherapist and patient participant interviews

We interviewed 11 participants from the intervention arm at the end of the 16-week treatment period and all 11 physiotherapists delivering the intervention and 3 site principal investigators. The findings of the participant interviews are presented in supplementary file 4.

Based on the interviews participants and physiotherapists reported that they found the intervention acceptable and that it had a place in future ways of working. Physiotherapists found the self-management training particularly helpful as evidenced by them adopting this approach in their usual practice. However, there was also evidence of this not being fully embedded in their treatments as they reported this approach working best for participants with certain characteristics, such as being more motivated.

Participants reported that TRAK MSK was easy to use and only required basic computing skills. They identified that it helped increase their knowledge and understanding of their condition, to set goals and select exercises and self-direct their treatment. They also identified some specific requirements to get full benefit from TRAK MSK, such as willingness to undertake remote treatment, confidence in their ability to exercise and digital literacy. Some expressed concerns around feelings of anxiety and loneliness with an online approach, the need to seek greater feedback and reassurance and being ill equipped to select their exercises and goals. They reported more satisfaction with TRAK MSK when physiotherapists took a collaborative approach.

#### Intervention fidelity and adherence

Data related to the self-reported self-management concepts used by practitioners are detailed in Table 6. The most used concepts discussed were that of goal setting (83%) and Problem-solving (81%), while other concepts about support and taking action, were less well used in the session. On average TRAK MSK participants attended 3 online consultations. All usual care consultations received in person.

**Table 6:** Practitioner sessions (adherence) and Self-Management concepts used

**Length of Sessions**	Mean length (mins)
Session 1/Initial Assessment	42.8
Sessions 2–5	22.2
Session duration	30.3
Number of total sessions per participant	(*N* = 28)
1	5
2	11
3	7
4	2
5	3
Total number of interventions	71
Core Principles of self-management	Number of times used
Personalised goals	59/71
Problem-Solving	58/71
Reflection on mastery experiences	53/71
Personal communities	22/71
Self-Discovery	57/71
Knowledge of self and condition	50/71
Taking Action	37/71

## Discussion

The overall aim of this study was to evaluate the feasibility and acceptability of delivering the TRAK MSK digital physiotherapy supported self-management intervention for people with joint pain compared to usual care. We demonstrated feasibility (green traffic light progression criteria) for recruitment and randomisation and (amber traffic light progression criteria) for follow-up rates and collection of health resource use data. Interviews with participants and physiotherapists suggested the intervention was acceptable and had a place in future ways of working.

We propose some modifications in recruitment and online study processes linked to consent and accessing the study website, to ensure targets are met in a future definitive trial. These recommendations were supported by the Trial Steering Committee. At our study sites, most participants were identified in person at clinic or from waiting lists. Both routes had strengths and weaknesses, for example, contacting people on the waiting list reached large numbers but had a lower conversion to study uptake. In person recruitment had a higher uptake but was slower and reached fewer people. Therefore, we would encourage sites to adopt multiple recruitment strategies such as in person recruitment from clinics, waiting lists, posters and flyers in clinical areas [51–53]. In addition, our healthcare resource use data indicated that most musculoskeletal care is delivered in primary care. Therefore, other options for recruitment need to be considered such as pharmacies, health centres and general practice surgeries.

In a future trial we would need to increase the sample size to account for the drop-out rate related to online recruitment and the three steps potential participants need to complete before randomisation: (1) complete expression of interest form, (2) sign consent form and (3) complete online baseline questionnaires. We will also work with members of the public to make sure we are not overburdening them with online forms. For example, we found some redundancy of items in the healthcare resource use measure. Finally, based on email queries we received we would ensure that the URL for the study website, is optimised for google searching. The URL used for the current study was like another trial, which appeared higher in google searches. Some participants didn’t complete their registration in one sitting and found it difficult to find our website when they returned. This also potentially impacted on follow-up rates of questionnaire completion at the end of the intervention.

Our post intervention follow-up rate was 60%, which is comparable to other digital interventions in this field lasting 12 weeks, where attrition ranges from 11–39% [12]. To improve retention, fewer patient withdraws from the intervention arm is required. Interview data revealed that some physiotherapists withdrew patients when they felt the online format was unsuitable opting for in person treatment instead. This may be related to the high number of participants recruited directly from waiting lists, suggesting that future studies may require more robust screening procedures. One solution proposed by both physiotherapists and participants was to have an initial face to face consultation before moving online.

Since this trial was designed as an external randomised feasibility trial rather than a definitive trial, as per guidance, our interpretation of the secondary outcomes is focussed on the direction of effect rather than the magnitude of any between group differences. When considering the direction of effect evidenced in PROMs at follow up, scores on MSK-HQ, EQ-5D 5L and pain self-efficacy score (PSEQ) were higher after intervention receipt whilst pain scores (NRS) were lower. The reported differences were greater than the published minimum clinically important differences [54–57] for these measures and aligned to the programme theory for TRAK MSK. This suggests that engaging in the intervention may result in greater confidence to manage pain and resultant improvements in function. In those who received usual care, there were also improvements in the MSK-HQ which were greater than the published minimal clinically important difference for this study population. In the usual care group, changes in other measures fell outside the published ranges for minimal clinically important differences. This suggests that although participants experienced some benefit from the physiotherapy they received, they did not gain increased confidence in managing their pain or exercise.

In the TRAK MSK group at follow up, scores on EQ5D-VAS, GAD7 and PHQ9 were marginally changed (indicating worse health on the day, slightly greater anxiety and slight increases in depression). However, none of these differences could be considered within the published ranges of the minimal clinically important differences for these PROMs [58, 59]. Based on these findings the decision to progress with a superiority trial design would be discussed with PPI, trial methodologists and clinicians.

Health resource use data were feasible to collect. Primary care was the most common location for care so the emphasis of the RUM should be primary/community care. Day case appointments may be causing confusion as the day cases reported appeared more likely to be outpatient appointments, so the instructions need to be clearer. Medications were well reported, although several respondents did not know the dosage of the medication. In a full trial expert opinion may be needed to determine the most likely dosages for medications for musculoskeletal pain. Also, a few respondents reported taking medication ‘as needed’, so PPI input would be useful to interpret the frequency of use in these instances.

Productivity data reporting was mixed, employment was well reported, as was impact on activities of daily living, however impact on productivity was variably reported, with some questions answered by all participants, whereas some were missed, possibly if they were not felt relevant to the participant. Given approximately half the participants at baseline reported that musculoskeletal pain had impacted their ability to work, this appears to be an important resource item to capture in a future trial.

Both the EQ-5D-5L and ICECAP-A were completed by all the individuals that participated in the 16-week follow-up. In a future trial involving health economics evaluation, the EQ-5D should be included as it is the more commonly used measure. However, the ICECAP-A, which takes a broader approach by assessing overall wellbeing, also appears suitable for this population based on its high completion rate in our data.

Some physiotherapists perceived that the TRAK MSK intervention was best suited to a particular type of patient. They found it easier to deliver the treatment to those that they thought would benefit most, such as those that were highly motivated, had high digital and health literacy, and were physically active and less anxious. Alongside this some TRAK MSK participants were concerned about digital literacy, willingness to be treated remotely, confidence to exercise and isolation and anxiety, all of which aligns with previous literature [60]. To address these challenges, it is essential to support the development of participants’ digital health literacy skills. This will enable them to effectively use digital tools to communicate, navigate healthcare information and make informed decisions around their health and care [61]. Consequently, future physiotherapist training for self-management support should include components that build knowledge, confidence and skills needed to integrate digital health literacy coaching into TRAK MSK consultations. The TRAK MSK website contained tools for exercise prescription and information sources. If digital health literacy is better integrated into self-management support, patients may be better equipped to use resources tailored to their needs. This could reduce the need to maintain bespoke resources on TRAK MSK website.

Participants attended an average of 3 online consultations, which was in line with other online rehabilitation intervention trials and met pre-defined levels of adherence [62]. However, there is some evidence that the self-management approach was not fully embedded in the practice of the physiotherapists delivering the TRAK MSK intervention. The fidelity assessment indicated that key self-management principles were not well used, for example, there was little evidence of physiotherapists supporting people to access their support network and to take action to do more in their everyday lives and reflect on this. Therefore, wrap around support for the physiotherapists such as drop-in sessions with Bridges trainers and ongoing access to exemplar videos and intervention prompts could help to improve fidelity, consistency of delivery and embed in practice [63, 64].

### Limitations

Follow-up was limited to 16 weeks, so the longer-term feasibility and acceptability are unknown. Reasons for practitioners withdrawing participants from the intervention were not recorded but these insights might have been important in the design of a future RCT. Participants were not able to access the online questionnaires for nearly 2 weeks due to software issues outside of the control of the study team. However, participants were still receiving reminders to complete their forms which they could not do and may have resulted in a poor experience and help explain reduced completion rates at 16 weeks.

We recruited through self-referral via social media adverts and clinicians identifying participants in clinic and waiting lists. In the future we would also include flyers and posters in the community so we access those without the internet and who may be more disadvantaged. Participants recruited to this trial were not as ethnically or socioeconomically diverse as we would have liked despite providing options for increasing accessibility to the trial i.e. loan of device, telephone support. This would need to be addressed in the design of a future trial [65]. Options to increase diversity include involving more community-based groups in our recruitment and printing out study material in more languages.

The fidelity assessment was based on physiotherapist self-report and thus prone to reporter bias. Assessment of the effectiveness of the physiotherapist training was not evaluated. In a future trial fidelity assessment would be carried out by audio recording 2 consultations for 2 participants and evaluated against a fidelity checklist by a member of the Bridges team. Data from this project will be used to refine the current checklist.

Usual care participants were not interviewed as part of the process evaluation. This would have helped define the treatment these participants received, as this was in a state of change post the covid 19 pandemic. This would be recommended as part of the process evaluation in a future trial.

### Strengths

We met targets for recruitment, randomisation and follow-up rates set out by pre-determined progression criteria. We have evaluated the feasibility of collecting health resource use data, which is important as this has been highlighted as a gap in previous RCT’s in this field [66]. Our online processes for study enrolment, consent and completion of patient-rated outcomes were successful and reduced burden for NHS sites delivering the trial. However, we would recommend a multi-layered approach to recruitment, incorporating clinical and self-referral to counteract inherent selection related to those that are most motivated in enrolling [12]. The process evaluation interviews with patient participants and practitioners provided greater understanding of our findings and identification of key modifications required for a future trial. Our data on intervention costs suggest that TRAK MSK costs less to deliver than usual care.

## Conclusion

The findings of this external feasibility trial suggest that progression to a full trial with modifications is recommended. This is based on our achieving all three prespecified progression criteria at green or amber levels and participants reporting that the intervention was acceptable. Receiving a red criterion would have halted progression to full trial. There was evidence that supported self-management skills were not fully integrated into practice but ways of improving this have been identified, such as drop-in sessions and access to exemplar videos. Future physiotherapist training for self-management support should include components that build the knowledge and skills needed to integrate digital health literacy coaching for patients into TRAK MSK consultations. The findings of this feasibility trial will guide the design of a future definitive trial. This needs to be carried out in collaboration with PPI representatives, clinicians, patient advocates and trial methodologists and must incorporate strategies to improve the inclusion of underserved groups.

## Supplementary Information

Supplementary Material 1.

Supplementary Material 2.

Supplementary Material 3.

Supplementary Material 4.

## Data Availability

The datasets used and/or analysed during the current study are available from the corresponding author on reasonable request.

## Abbreviations

EQ-5D 5L: EuroQol 5 dimension 5 level instrument
GAD-7: General Anxiety Disorder Scale
GCSE: General Certificate of Secondary Education
ICEpop: Capability Measure for Adults ICECAP-A
MSK-HQ: Musculoskeletal-HQ health status questionnaire
MSK-PA: Musculoskeletal Physical Activity sub-scale
PSEQ: Pain self-efficacy score
PHQ-9: Patient Health Questionnaire for Depression
GAD-7: General Anxiety Disorder Scale
MRC: Medical Research Council
NHS: National Health Service
N: Number
NRS: Numeric rating scale
PPI: Patients and public involvement
PROM: Patient rated outcome measure
RCT: Randomised controlled trial
RUM: Resource use measure
SD: Standard deviation
TRAK MSK: TRAK Musculoskeletal
