# Supplementary material for: TRAK MSK: a randomised external feasibility trial and process evaluation of a digital physiotherapy supported self-management programme for people with musculoskeletal pain
Source: BMC Musculoskelet Disord. 2026 Jul 4;27:799. doi: 10.1186/s12891-026-10170-y (PMC13613736; doi:10.1186/s12891-026-10170-y)
Supplement: Supplementary file 1 [file 12891_2026_10170_MOESM1_ESM.docx]

# Please tell us about the healthcare you have received in the last three months for muscle, joint, bone or soft tissue (musculoskeletal) pain.

**Please circle ‘yes’ or ‘no’ for each item. If you answer ‘yes’ to any of them, please tell us how many times you used the service in the last three months.**

In the last three months, have you visited or spoken to your GP or practice nurse because of musculoskeletal pain?

|  | **Please circle answer** | **Number of appointments/visits/ phone calls** |
| --- | --- | --- |
| GP face-to-face appointment at surgery | Yes No |  |
| Spoke to GP on the telephone | Yes No |  |
| Spoke to GP online/video call | Yes No |  |
| Practice nurse face-to-face appointment at surgery | Yes No |  |
| Spoke to practice nurse on telephone | Yes No |  |
| GP out-of-hours appointment | Yes No |  |
| District nurse home visit | Yes No |  |
| Called NHS 111 | Yes No |  |

In the last three months, have you had any physiotherapist or occupational therapist appointments in the community? (do not include hospital appointments or private appointments in this section)

| **Description** | **Please circle answer** | **Number of appointments** |
| --- | --- | --- |
| Physiotherapist appointment at a GP surgery or community centre e.g. MSK centre | Yes No |  |
| Occupational therapist appointment at the GP surgery or a community centre | Yes No |  |
| Podiatrist appointment at a GP surgery, or a community centre | Yes No |  |
| Other appointment at the GP surgery or community centre, please specify healthcare professional seen: | Yes No |  |
|  |  |  |
|  |  |  |

In the last three months have you visited a private healthcare professional or alternative therapy practitioner for your musculoskeletal pain?

| **Description** | **Please circle answer** | **Number of appointments** | **How much did you pay per appointment?** |
| --- | --- | --- | --- |
| Physiotherapist | Yes No |  |  |
| Occupational therapist | Yes No |  |  |
| Osteopath | Yes No |  |  |
| Chiropractor | Yes No |  |  |
| Podiatrist | Yes No |  |  |
| Massage therapist | Yes No |  |  |
| Acupuncturist | Yes No |  |  |
| Aromatherapist | Yes No |  |  |
| Other appointment, please specify: | Yes No |  |  |
|  | |  |  |
|  | |  |  |

In the last three months, have you had any outpatient appointments at hospital because of your musculoskeletal pain? (do not include scans or tests in this section)

| **Description** | **Please circle answer** | **Number of appointments** |
| --- | --- | --- |
| Trauma and orthopaedics | Yes No |  |
| Pain management | Yes No |  |
| Rheumatology | Yes No |  |
| Physiotherapy | Yes No |  |
| Occupational Therapy | Yes No |  |
| Podiatry | Yes No |  |
| Orthotics | Yes No |  |
| General medicine | Yes No |  |
| General surgery | Yes No |  |
| Spinal surgery service | Yes No |  |
| Spinal Injuries | Yes No |  |
| Sport and Exercise Medicine | Yes No |  |
| Rehabilitation Service | Yes No |  |
| Other, please specify   | Yes No |  |

In the last three months, have you any tests or scans at hospital because of your musculoskeletal pain?

| **Description** | **Circle answer** | **Number of times** |
| --- | --- | --- |
| X-ray | Yes No |  |
| CT scan | Yes No |  |
| MRI scan | Yes No |  |
| Blood tests | Yes No |  |
| Other, please specify | Yes No |  |
| Other, please specify | Yes No |  |

In the last three months, have you attended hospital as a day case because of your musculoskeletal pain? If ‘yes’ please specify the healthcare service under which you attended.

| **Description** | **Circle answer** | **Number of times** |
| --- | --- | --- |
| Day case | Yes No |  |
| Please specify service |  |  |
| Please specify service |  |  |

In the last three months have you been attended by a paramedic or visited A&E due to your musculoskeletal pain?

Please circle ‘yes’ or ‘no’ for each item. If you answer ‘yes’ to any of them, please tell us how many times you used the service.

| **Description** | **Circle answer** | **Number of attendances** |
| --- | --- | --- |
| Attendance by paramedic/ambulance at home, not transferred to hospital | Yes No |  |
| Attendance by paramedic/ ambulance at home and transferred hospital | Yes No |  |
| Attended a walk-in clinic due to musculoskeletal pain | Yes No |  |
| Attended accident and emergency due to musculoskeletal pain | Yes No |  |

In the last three months have you been admitted to hospital due to your musculoskeletal pain?

Please circle ‘yes’ or ‘no’ and note the number of admissions. If you answer ‘yes’, please note for each admission if you were admitted through accident and emergency, and the total number of days in hospital per admission.

| **Description** | **Circle answer:** |  |
| --- | --- | --- |
| Admitted to hospital | Yes No | Total number of admissions:   |
| Admission one | Admitted from accident and emergency, please circle:  Yes No | Total number of days in hospital:   |
| Admission two | Admitted from accident and emergency, please circle:  Yes No | Total number of days in hospital:   |
| Admission three | Admitted from accident and emergency, please circle:  Yes No | Total number of days in hospital:   |
| Admission four | Admitted from accident and emergency, please circle:  Yes No | Total number of days in hospital:   |

In the last three months, what medicines and dressings have you used for your musculoskeletal pain and how did you pay for them? Please include prescriptions, non-prescription items (over the counter), homeopathic remedies and herbal medicines.

| **Pain killers:**  Yes No  **Name:** | Dose per day (if relevant) | How long have you been taking this medicine? | Was this an NHS prescription? |
| --- | --- | --- | --- |
|  |  |  | Yes No |
|  |  |  | Yes No |
|  |  |  | Yes No |
| **Anti-inflammatory drugs:**  Yes No  **Name:** | Dose per day (if relevant) | How long have you been taking this medicine? | Was this an NHS prescription? |
|  |  |  | Yes No |
|  |  |  | Yes No |
|  |  |  | Yes No |
| **Gels/creams:**  Yes No  **Name:** | Dose per day (if relevant) | How long have you been using this gel/cream? | Was this an NHS prescription? |
|  |  |  |  |
|  |  |  |  |
|  |  |  |  |
| **Sleeping pills:**  Yes No  **Name:** | Dose per day (if relevant) | How long have you been taking this medicine? | Was this an NHS prescription? |
|  |  |  | Yes No |
|  |  |  |  |
| **Anti-depressants**  Yes No  **Name:** | Dose per day (if relevant) | How long have you been taking this medicine? | Was this an NHS prescription? |
|  |  |  | Yes No |
|  |  |  |  |
| **Dressings**  Yes No  **Name:** | How many times do you change these dressings | How long have you been using this dressing? | Was this an NHS prescription? |
|  |  |  | Yes No |
|  |  |  |  |
| **Other**  Yes No  **Name:** | Dose per day (if relevant) | How long have you been taking this medicine or used these dressings? | Was this an NHS prescription? |
|  |  |  | Yes No |
|  |  |  | Yes No |
|  |  |  | Yes No |

# We would also like to know what musculoskeletal pain has cost you, and your friends and family.

In the last three months, have you (or friends and family on your behalf) bought items such as braces or aids, a new bed or mattress, chair, or any other products or equipment because of musculoskeletal pain? Please list the item below and estimate the cost to the nearest pound.

| **Item bought:** | **Cost** |
| --- | --- |
|  | £ |
|  | £ |
|  | £ |
|  | £ |
|  | £ |
|  | £ |
|  | £ |

Please indicate your employment status for the last three months?

| **Description** | **Please put a tick next to the description that most closely matches your circumstances:** |
| --- | --- |
| Full-time employment |  |
| Part-time employment |  |
| Unemployed and looking for work |  |
| Unable to work | Due to musculoskeletal pain, please circle:  Yes No |
| Volunteer |  |
| Job training/apprentice |  |
| Student |  |
| Retired | Have you taken early retirement due to musculoskeletal pain, please circle:  Yes No |
| Full-time carer |  |

If you are in employment, please answer the following questions for the last three months:

| **Description** | **Please circle:** | **Number of days:** |
| --- | --- | --- |
| Have you taken any days off sick from work due to musculoskeletal pain? | Yes No |  |
| Have you used paid holiday time from work due to musculoskeletal pain? | Yes No |  |
| Have you taken unpaid leave from work due to musculoskeletal pain? | Yes No |  |
| Have you attended work, but felt your ability to work has been impacted by musculoskeletal pain? | Yes No |  |
| Have you had to make up time at work due to musculoskeletal pain? | Yes No |  |

Over the last three months has your musculoskeletal pain stopped you doing your normal daily activities such as education and caring responsibilities?

| **Description** | **Please circle:** | **Number of days:** |
| --- | --- | --- |
| Have you been unable to do housework or food shopping? | Yes No |  |
| Have you been unable to care for children or a relative? | Yes No |  |
| Have you been unable to attend school, college or university due to musculoskeletal pain? | Yes No |  |

Over the last three months has your musculoskeletal pain stopped you participating in leisure or sporting activities?

| **Description** | **Please circle:** | **Number of days:** |
| --- | --- | --- |
| Have you been unable to attend adult education classes or do your hobbies? | Yes No |  |
| Have you been unable to participate in regular exercise or sporting activities? | Yes No |  |
| Have you been unable to attend social events? | Yes No |  |

In the last three months have you needed support from a friend or family member due to your musculoskeletal pain? (if support was needed to attend healthcare appointments please report this in the next question)

| **Description** | **Please circle:** | **How many hours per week?** |
| --- | --- | --- |
| Childcare or to care for a relative | Yes No |  |
| Housework/laundry | Yes No |  |
| Shopping | Yes No |  |
| Providing transport/taking you out | Yes No |  |
| Looking after pets | Yes No |  |
| Other, please specify | Yes No |  |

In the last three months have you needed support from a friend or family member to attend healthcare appointments related to your musculoskeletal pain?

| **Description** | **Please circle:** | **How many hours per week?** |
| --- | --- | --- |
| To accompany you to healthcare appointments | Yes No |  |
| Transport to appointments | Yes No |  |
| To provide childcare or to care for a relative while you attend an appointment | Yes No |  |
| Other, please specify | Yes No |  |

**We would like to understand the impact of COVID-19 on your access to everyday activities**

In the last three months, have COVID-19 restrictions impacted you attending any healthcare appointments or taking part in any activities, excluding employment?

|  | **Please circle:** | **Number of appointments/activities** |
| --- | --- | --- |
| Have COVID-19 restrictions prevented you from attending any healthcare appointments in the last three months? | Yes No |  |
| Have you cancelled any healthcare appointments due to concerns about COVID-19 in the last three months? | Yes No |  |
| Have you changed to online activities due to COVID-19? | Yes No |  |
| If you have the option of these activities being online or face-to-face in the future which would you prefer? | **Please circle:**  Online  Face-to-face | |
| Have you tested positive for covid-19? | Yes No | |
| If you have had covid-19 has this made your musculoskeletal pain worse? | Yes No | |
| Have you been referred to a specialist covid-19 clinic? | Yes No | |
