# Supplementary material for: TRAK MSK: a randomised external feasibility trial and process evaluation of a digital physiotherapy supported self-management programme for people with musculoskeletal pain
Source: BMC Musculoskelet Disord. 2026 Jul 4;27:799. doi: 10.1186/s12891-026-10170-y (PMC13613736; doi:10.1186/s12891-026-10170-y)
Supplement: Supplementary file 2 [file 12891_2026_10170_MOESM2_ESM.docx]

**Interview schedule for patient participants**

1. How would you describe the TRAK study to a friend?
2. What did you expect from taking part in the TRAK study? – and did it meet your expectations?
3. Do you feel you have learned anything about your condition by taking part in the TRAK study? If so, what?
4. How do you feel about the online consultations in meeting your healthcare needs?

- What did the online consultations with physiotherapists give you/ What did you take from them?
- How many sessions did you have with the physiotherapist?

5. What do you understand about how good nutrition can help you manage your condition?

- Did nutrition come up at all in your consultations with physiotherapists?
- Can you tell me more about your discussion on it?

6. In your interactions with physiotherapists and using the myjointpain website did you receive or find the exercises you were expecting?
7. What did you find easy or difficult to navigate on the myjointpain website?
8. You might have noticed that the physiotherapist you worked with would have tried to encourage you to make as many choices for yourself as possible.

- How did that feel to make decisions for yourself, instead of being told directly what to do by a healthcare professional?

9. Whilst taking part in the TRAK study were you able to set your own goals?

- Did you find you met any of your goals?
- How useful is setting goals in the management of your condition? [probe – how, why, why not etc.]

10. Do you know which exercises and activities are best (or work best) for you, to help you manage your condition?

- Has taking part in the TRAK study helped you develop that knowledge?
- In what way?

11. Do you feel you know what to do if the pain you experience gets worse?

- Has taking part in the TRAK study helped you with that at all? Can you give an example?

12. Do you feel you know how to manage the pain or any discomfort you experience while exercising?

- Has taking part in the TRAK study helped you with that?

13. Do you feel able and confident to design your own activity and exercise plan to help you manage your condition?

- Has taking part in the TRAK study helped you develop that ability?

14. Is there anything you still don’t feel confident about in managing your condition?

- Can you say why you’re not confident of that? ( if applicable)

15. Did you surprise yourself in any way during taking part in the TRAK study?

- Tell me about that

16. Do you see having an online consultation as part of supporting the environment by reducing our carbon footprint?

- Does supporting the environment in this way make you feel that online consultations like TRAK are more or less acceptable?

**Interview schedule for Physiotherapists**

**Background of individual**

1. Please can you tell me about your experience of working on clinical trials?
2. Please can you tell me about your experiences of working with clinical trial databases / websites?
   - PROMPT – If the interviewee has not had any prior experience of working with clinical trial databases, ask about general experience of working with databases and about approach to record keeping in the NHS/ why trial database is critical.
3. What expectations did you have about using the TRAK database?

- What advantages/challenges did you envisage?

4. How was it to do online registration for the trial?

- How did this impact on the rest of your daily/weekly work
- What type of impact was there on patients?

5. How much online physiotherapy consultation do you carry out in your job? How do you find practising physiotherapy in an online space? [probe dependent on level of exposure and experience before and through covid]
6. What do you feel are the benefits and challenges of online consultations in general and in reference to TRAK?
7. How do you find being a TRAK intervention physiotherapist compared to usual work?

- How long have you been involved in TRAK?
- How long did it take to get used to the process?
- Were there any challenges for you in TRAK? What were they?
- Compared to the early days of the project, what is it like now?
- What do you think about the timeframes you’re given for work on TRAK – per patient, per day, over whole project?

8. How does TRAK differ from usual care for patients?

- In what way/s are things different? How many elements of care do you think are different to usual care?
- What benefits are there in Trak vs Usual care for you as physio?
- Any disadvantages?
- What advantages/ disadvantages are there for patients in your view?

9. What type of cases work best as TRAK for you? For patients?

- Any types of case that doesn’t work so well for you or for patients?

10. How does using TRAK impact on your day to day work now?

- Has this changed from the start of the study?
- Are there any problems with ‘ fit’ into the overall timetable of your work?
- Do you plan and divide your work differently because of TRAK?

11. How useful and successful is TRAK in engaging patients now compared to the start of the project?

- How do patients react to TRAK process?
- Do some patients engage better than others – which ones?
- Overall, what do you think was the impact on/engagement with patients during coaching? What do they tell you about the process?

**ii) Using myjointpain website and self-management approach**

12. What was your impression of how patients engaged and got on with using the myjointpain website?
13. How did you find supporting patients to navigate an online resource? Did you find patients needed support from you to access or use any aspects of the online resource?
14. What changes or improvements would you like to see to the myjointpain website?
15. Bringing those two elements together - what do you feel meeting with patients and their use of the website offers them in terms of managing their condition?
16. How did you find implementing self-management strategies in this form of coaching session/interaction?

- What worked well from your perspective?
- Anything that didn’t work so well?
- What for you, are the strengths of a self-management approach in general?
- Any limitations of using a self-management approach, can you give details or an example?

17. When you’ve used a self-management approach in a session – how do you record that?

- Same as before or not? Can you give details?

18. How did you find the Bridges training and the training you had to take part in the study?
19. Do you feel that interventions like TRAK – or any components of it, have a future in the delivery of physiotherapy? [probe – how, why]
