# Supplementary material for: TRAK MSK: a randomised external feasibility trial and process evaluation of a digital physiotherapy supported self-management programme for people with musculoskeletal pain
Source: BMC Musculoskelet Disord. 2026 Jul 4;27:799. doi: 10.1186/s12891-026-10170-y (PMC13613736; doi:10.1186/s12891-026-10170-y)
Supplement: Supplementary file 3 [file 12891_2026_10170_MOESM3_ESM.docx]

**Supplementary File 3**

*Healthcare resource use*

Primary care data was well-reported at both time points, with no missing data at baseline, although 3 participants did not complete the RUM at 16 weeks (TRAK N=20, usual care N=22). At baseline primary care was the most reported healthcare resource used and by 16 weeks there was much less healthcare resource use reported (Table 1).

Table 1 Primary/ community care healthcare resource use

|  | **TRAK (N=36)**  **Baseline** | **Usual Care (N=37)**  **Baseline** | **TRAK (N=20)**  **At 16 weeks** | **Usual Care (N=22)**  **At 16 weeks** |
| --- | --- | --- | --- | --- |
| **GP face-to-face appointment** | 17 (47.2%) | 16 (43.2%) | 1 (5.0%) | 5 (22.7%) |
| **GP telephone appointment** | 11 (30.6%) | 12 (32.4%) | 0 | 5 (22.7%) |
| **GP online/video call** | 0 | 0 | 0 | 0 |
| **Nurse face-to-face appointment** | 0 | 4 (10.8%) | 1 (5.0%) | 1 (4.6%) |
| **Nurse telephone appointment** | 0 | 3 (8.1%) | 0 | 0 |
| **GP out-of-hours** | 0 | 1 (2.7%) | 0 | 0 |
| **District nurse visits** | 0 | 0 | 0 | 0 |
| **Calls to NHS 111** | 0 | 0 | 0 | 0 |
| **Physiotherapist appointments** | 4 (11.1%) | 7 (18.9%) | 3 (15.0%) | 4 (18.2%) |
| **Occupational therapy appointments** | 0 | 0 | 0 | 0 |
| **Podiatrist appointments** | 0 | 1 (2.7%) | 0 | 0 |
| **Other healthcare appointments in primary care** | 4 (11.1%) | 1 (2.7%) | 0 | 0 |

Hospital appointments were not commonly reported at either time point. Outpatient appointments at baseline in general medicine (N=5, 6.8%), rheumatology (N=4, 5.4%), and trauma and orthopaedic appointments (N=4, 5.4%) were most reported, with additional appointments reported in pain management, physiotherapy, general surgery and rehabilitation. At 16 weeks physiotherapy was most commonly reported (N=10, 23.8%). There were no appointments reported for occupational therapy, podiatry, spinal surgery, spinal injuries, and sport and exercise medicine at either time point. There were no A&E attendances due to MSK pain, nor ambulance attendances. There were N=3 (4.1%) attendances to walk-in clinics at baseline, and none at 16 weeks.

The most common form of imaging was X-rays (N=13, 17.8%), although CT scans and MRIs were also reported in both arms at baseline. Blood tests were performed for N=13 (17.8%) participants. At 16 weeks, 3 respondents had X-rays (7.1%), and 2 people had blood tests (4.8%), no CT scans or MRI scans were reported

Oral painkillers were most reported for MSK pain (N=64, 87.7% at baseline; N=35, 83.3% at 16 weeks), with a number of participants taking two (N=31, 42.5% at baseline; N=20, 47.6% at 16 weeks). Paracetamol and ibuprofen were most commonly taken, with Co-codamol, Naproxen, Tramadol, Codeine Phosphate use reported as well. Co-drydamol, dihydrocodeine, fentanyl, MST, MXL, Oramorph, Oxycodone and Zomorph use was not reported by any participants. Dosage was not always known, and for a number of participants, medication was used as needed. Gel painkillers were next most commonly reported (N=39, 53.4% at baseline; N=16, 38.1% at 4 months) with N=7 at baseline (9.6% baseline; N=2, 4.8% at 4 months) using two gel painkillers. Ibuprofen was used most often, followed by Voltarol, Capsaicin and Diclofenac (figure 1). No participants reported using patch painkillers at either time point.

Figure 1-Oral and gel/cream medications

The overall cost analysis for the TRAK trial is shown in Table 2. Given the small numbers of respondents at each time point it is not possible to draw any results from these inputs. The costs are likely to be skewed by the small numbers of day case attendances which can attract higher costs related to procedures. It is possible that the day cases reported are more likely to be outpatient appointments.

Table 2 Cost Analysis

|  | **TRAK - mean (SD)** | **Usual Care - mean (SD)** |
| --- | --- | --- |
| **Baseline - NHS** | £106.94 (£132.60) | £121.53 (£164.07) |
| **4 Month - NHS** | £39.15 (£101.99) | £149.98 (£269.18) |
| **Baseline – Private costs** | £104.69 (£175.49) | £215.29 (£504.93) |
| **4 Month – Private costs** | £12.08 (£25.11) | £66.21 (£154.81) |

## *Productivity Impact*

Levels of employment were reported for all participants, with the majority of participants in full-time employment (N=35, 47.9% at baseline), followed by retired (N=20, 27.4%). The questions on impact on productivity were not well-reported, with a large proportion of non-responses. Three respondents had taken early retirement due to MSK pain (N=3, 4.1% at baseline; N=10, 23.8% at 16 weeks). Some respondents had taken sick leave due to MSK pain (N=5, 6.8%), paid leave (N=7, 9.6%), and unpaid leave (N=2, 2.7%) at baseline. At 16 weeks fewer respondents reported an impact on productivity (taken sick leave – N=1, 2.4%; paid holiday – N=2, 4.8%; no unpaid leave was taken for MSK pain). Approximately half (N=34, 46.6% at baseline) of respondents at baseline, and 35.7% at 16 weeks (N=15) felt their ability to work had been impacted by MSK pain.

The impact of MSK pain on activities of daily life was well-reported, affecting participation in exercise or sport most (N=36, 49.3% at baseline; N=15, 35.7% at 16 weeks), then ability to do housework or shopping ( N=24, 32.9% at baseline; N=6, 14.3% at 16 weeks), as well as hobbies, attending social events, and being unable to care for children or a relative. Respondents required support for housework/laundry (N=20, 27.4% at baseline; N=6, 14.3% at 16 weeks), shopping (N=19, 26.0% at baseline; N=4, 9.5% at 16 weeks), as well as caring responsibilities, providing transport and looking after pets. Support from families and friends to attend healthcare appointments was only reported by a few respondents (N=4, 5.5% at baseline; N=1, 2.4% at 4 months). COVID-19 restrictions did not impact healthcare appointments, 3 people were unable to attend appointments at baseline, and 1 person at 16 weeks due to COVID-19.
