# Supplementary material for: TRAK MSK: a randomised external feasibility trial and process evaluation of a digital physiotherapy supported self-management programme for people with musculoskeletal pain
Source: BMC Musculoskelet Disord. 2026 Jul 4;27:799. doi: 10.1186/s12891-026-10170-y (PMC13613736; doi:10.1186/s12891-026-10170-y)
Supplement: Supplementary file 4 [file 12891_2026_10170_MOESM4_ESM.docx]

Supplementary File 4

*A table of key themes and illustrative quotes are provided in Table 1*

*Physiotherapist interviews*

**Theme 1: Comparison with usual care & changes to practice.**

When compared with usual care, the benefits that TRAK MSK brought to support self-management and patient choice were clear. Physiotherapists overwhelmingly reported a clear commitment to the TRAK MSK methodology, highlighting that the intervention had a role to play in a supported self-management approach to physiotherapy by giving patients the ability to take control of their rehabilitation. Whilst there were some reported difficulties around time management and the online nature of the consultation, the benefit it brought was apparent. Rather than prescribing a management programme, physiotherapists described TRAK MSK as enabling patients to take more ownership of what their rehab looks like. Indeed, the benefit that the intervention brought to consultations can be seen by physiotherapists reporting that they had begun adapting their approach by adopting elements of the methodology into their usual practice.

**Theme 2: The TRAK MSK approach; training, implementation & usability**

The Bridges training received by the physiotherapists was regarded as particularly beneficial. Whilst some reported undergoing some form of self-management training, the content and approach offered during the Bridges training was seen as specifically innovative, with accounts such as “the trainings were really insightful well led well and engaging” common. They also found the approach relatively straightforward to implement into their consultations, with the online medium facilitating this by allowing the physiotherapists to have prompts for further questioning.

During the consultations, physiotherapists introduced TRAK MSK in a variety of ways. Whilst some took an active role, going through the website with the patient, guiding them through the different exercises and working through goals and potential plans together, others took a relatively hands-off approach, allowing the patient to browse independently and set their own plan and goals. When reflecting on the usability of the website, it was deemed straightforward to navigate with a comprehensive ‘suite of exercises’ that were verified by physiotherapists that ultimately supported patient autonomy and self-management.

**Theme 3: Online methodology**

The digital element of TRAK MSK was found to be useful in supporting self-management. As one physiotherapist said, “having an online intervention promotes the ability for that patient to take control because they’ve got an array of exercises to look at and to progress themselves without having to have the physio talk them through everything … with a digital programme that they can explore themselves, it gives them greater ability to become confident at self-managing” [Staff interview 8].

The benefits that remote consultations brought around time constraints and travel issues were also discussed with some physiotherapists reflecting that this might encourage patients to maintain their rehabilitation more effectively.

Despite the perceived benefits that the online nature of TRAK MSK brought to patients, some physiotherapists struggled with remote consultations. Many discussed difficulties with not being able to “physically touch or feel the joints, and actually palpate to see what the problem is” [Staff interview 1] with them having to rely on verbal descriptions from patients instead. Some physiotherapists also reported building rapport with patients more challenging online, with reassurance difficult to develop during online consultations.

The digital literacy of some patients was also seen as a barrier to inclusion. For those patients who are not IT literate, there was some concern that it would “increase their anxiety about … the thought of doing it online [Staff interview 2].

**Theme 4: Patient requirements**

Whilst physiotherapists reported a strong commitment to the TRAK MSK methodology, the benefit was often framed around supporting the *right* patient; a patient who displayed self-motivation and high levels of health and digital literacy. Conversely, physiotherapists considered TRAK MSK as challenging for patients who sought a more hands-on approach; those who wanted a defined programme, clear guidance and to be directed by the physiotherapist.

The platform was also considered more suitable to specific forms of rehabilitation, with physiotherapists describing instances where the online nature of TRAK MSK was not appropriate. For example, “advancing hip osteoarthritis” where there was uncertainty around the patient “getting better without referring them onto specialist physios”.

Where this was deemed to be the case, physiotherapists reported taking the patient out of the trial to carry out a face-to-face consultation.

**Theme 5: Patient autonomy**

Physiotherapists reported high levels of patient satisfaction when the intervention was targeted at the *ideal* patient group. This ultimately had an impact on patients' ability to self-manage, increasing their feelings of control over their rehabilitation and developing their skills and confidence to manage their symptoms. However, where the intervention was considered challenging was when used with a seemingly unsuitable patient. Patients who were seeking a far more prescriptive approach to their rehabilitation were considered to have higher levels of anxiety and an increased need for reassurance when using the intervention.

**Theme 6: Future use of TRAK MSK**

Overwhelmingly, physiotherapists considered TRAK MSK as having a place in future ways of working when targeted at suitable patients. Whilst physiotherapists did not consider the intervention to require a shift in current working practice, reflecting that the website and training was beneficial for both practitioners and patients, many did suggest that an online consultation utilising TRAK MSK should follow an initial in-person consult. The flexibility that the intervention provides to certain cohorts of patients was seen as beneficial, with the key factor being around patient choice; that patients should be able to choose to undergo rehabilitation in an online format.

*Patients*

**Theme 2: TRAK MSK usability**

The patient participant group reported that TRAK MSK was easy to use overall, with only basic IT skills needed. The range and comprehensiveness of the exercises offered was particularly well regarded, enabling some patients to take control over their rehabilitation with only minimal input from the physiotherapist.

**Theme 3 & 4: Online methodology & patient requirements**

TRAK MSK users identified specific requirements that patients would need to gain the full benefit of the platform. In particular, a willingness to be treated remotely and confidence in their own ability to perform exercises without direction from the physiotherapist. Whilst this was considered to be beneficial for some patients, there were concerns around digital literacy requirements and the potential that this might “alienate a certain generation”. For some patients there was a strong resistance to the online nature of the intervention, expressing a preference for face-to-face consultations. Where this was discussed, there were reports of feelings of isolation and anxiety around digital technology.

**Theme 5: Patient autonomy**

Reports of increased knowledge and understanding around their rehabilitation were given by some patients which supported self-management. For these patients, they used TRAK to self-direct their rehabilitation and to set their own goals based on the available exercise information which ultimately led to increased sense of confidence in managing their own recovery. Others favoured a more passive approach to their rehabilitation and consequently found the expectation to pick their own exercises to support their recovery challenging. Here patients discussed seeking greater feedback and reassurance from physiotherapists, feeling ill equipped to be able to select the appropriate exercise plan and goals.

As discussed, physiotherapists similarly took different approaches to their consultations with patients with some taking on an active whilst others a more passive role. These differences in approach appeared to have a bearing on the engagement by patients, with some indication that patients found a more hands-off approach difficult and were more likely to display resistance to the overall approach. However, descriptions of physiotherapists approaching the consultation in a more collaborative manner, particularly in the initial consult, where together suggestions of exercises with a jointly worked through plan appeared to lead to greater satisfaction amongst patients.

**Theme 6: Future use of TRAK MSK**

When considering using the platform in the future, and potential roll out to other patient groups, users felt that a blended approach to treatment would be preferable, by combining initial face-to-face contact alongside subsequent utilisation of TRAK MSK.

**Table 1: Key themes with illustrative quotes**

| **Study theme** | **Data source** | **Illustrative quotes** |
| --- | --- | --- |
| **Theme 1**  **Comparison with usual care & changes to practice** | Physiotherapist | I think we’re still quite prescriptive in terms of erm the home exercises, so again it's the physiotherapists identifying exercises following the consultation and prescribing those exercises erm to try and match the patients goals erm and expectations. Erm, whereas through TRAK MSK with the intervention group having access to this library of exercises, erm with them taking more ownership of what their rehab looks like. So, whether that's a playlist of a certain exercise, erm a certain difficulty level for their body part that they're having issues with or whether it's taking individual exercises to make their own erm, programme for them to take forwards. Erm, so a bit less prescriptive [Staff interview 13]. |
|  |  | I think, erm, we, we’ve got into a bit of a habit with usual care that we’re … prescribing a management programme that they go away to carry out. whereas now and we’re focussing on, as much as we try, we do generally ask quite close questioning, what do you want to achieve and we’re goal setting but actually it’s looking at more of a holistic approach, asking questions, kind of what would make you feel more like you, what would success look like for you, really encouraging the active listening and rather than saying, here’s the exercises, I want them done three times a day, off you go, it’s, these are the, this is the exercise suite, you choose what suits you, what you feel would help you, have a little look through, ‘cause it’s putting the ownership on them a lot more as to what they would feel helps them and what works for them. [Staff interview 1]. |
| **Theme 2**  **The TRAK MSK approach; training, implementation & usability** | Physiotherapist | I found the training, really, really beneficial, um, the sort of Bridges um, aspect of stuff, really helped sort of, yeah, both with the TRAK intervention and also my own kind of practice as well. It opened my eyes to kind of, um, giving the patients a little bit more sort of freedom sort of around, what they decided to do, also that um, thinking about um, how much of a, an aspect or confidence and self-efficacy has on, on, on, how a patient does, with, with physio as well, in, in certain groups if you can increase that, or have work on that sort of self-efficacy, you actually take a lot more from physio than just by giving them monthly exercises, they might not feel confident to do [Staff interview 3]. |
|  |  | I think the Bridges is, is definitely really useful in clinical practice, I think it’s asking the questions we necessarily, haven’t necessarily asked a patient in the past before, so, exploring previous beliefs, um, and previous experiences... It’s definitely good for remote consultations, because you can write a little prompt note to yourself, go through it and being able to add that to their, their notes, it is helpful, it makes you prompt to ask them questions [Staff interview 4]. |
|  | Patient | I thought it was a fantastic website, it really, oh, it is a fantastic website, it’s just so comprehensive [Patient interview 1]. |
|  |  | It’s obviously, err, taught me how um, I can obviously, best sort of ease my, my pain, um, previously, I was sort of, probably using quite a lot of paracetamol to try and just deal with the pain and live with it. Um, whereas, now, there’s, I know that there’s um, exercises that I can do, err, and then obviously, just building up the strength within, um, um, the areas that I have, the issues, um, to hopefully make them, make them better and that’s kind of the process I, I’m going through at the moment. But I can see a difference, um, which is um, which is obviously, making me happy um, if I’m able, able to be a bit more um, active [Patient interview 3] |
| **Theme 3**  **Online methodology** | Physiotherapist | I think also from a situational point of view, sometimes people really benefit from not having to come into a department if they're, you know, working away a lot or they can't get time off work, erm I think that type of patient group can find it really helpful. At the moment I've got a patient that used to live in [place name] that now lives in [place name] erm and rather than handing over care or losing him, I see just him remotely via kind of video or telephone conversations just to make sure he's still on the right lines erm and guiding his rehab a little bit but lately it's kind of enabling, empowering him to carry on independently and slowly kind of making him more independent [Staff interview 14]. |
|  |  | Some of our patients, were too severe to be self-managed and I think that’s again, that’s a problem with, so, remote consultations are great, the problem is for patients that were picked, one needed to go back and see their consultant, cos they’d had some new injury. So, again, I couldn’t assess them. And, that’s, you know, a clinical risk that I actually need to see you, I need to do some tests on your knee or whatever, um, another one was waiting Rheumatology, cos they had chronic pain It was just, they, they didn’t, it didn’t work with the website [Staff interview 4]. |
|  |  | I think there’s, um, I think the value of kind of um, being in the same space as someone and that kind of reassurance that the patients get from that, is, is lost in the, the video consultations. I don’t think that’s just me, as a clinician, preferring that, I do feel that a lot of the feedback, we’re kind of having from patients, is that you would say, do all the kind of same reassuring and everything, say for like a knee or something but they, the kind of follow-up would be, “Well, could you actually just take a look at it?” And, I think that’s what kind of was missing in the kind of virtual stuff [Staff interview 11] |
|  | Patient | Occasionally, I get an ache in my hip, and, um, which I, which I don’t think it’s related to um, being worn out or anything, but I just thought, oh, I could always have a look at the hip ones, and see what they say, or you know. I, I, I just feel like I’ve got a little um, treasure trove of information, that you know, if I get any niggles, I can have, have a look. I mean, if it was anything really serious, I would go to a physio or whatever, an appointment. But if it was important, but I can at least have a look, and maybe get some pointers as to what’s going on, which I never had before. [Participant Interview 1]. |
|  |  | Having a suite of exercises, to, to learn and to understand, err, and, and to be able to you know, sort of go back to, err, as and when needed, err, has, has been a, been a, a great, great help [Patient interview 3] |
| **Theme 4**  **Patient requirements** | Physiotherapist | I think it’s a type of person, it has to be someone probably a bit more motivated, you know, we do get a lot of patients who need regular supervision who, who need to be told they don’t know what they want, they want to know how many repetitions, how many things, what to, what needs to be done, they need that prescription. Erm, whereas when you’ve got patients who are maybe a bit more mobile, encouraged, motivated to get back that they’re going to probably do better on it [Staff interview 1]. |
|  |  | You’ve gotta try and find a patient, that’s in that kind of that, that stage, where they want to change. Where it’s not gonna work, um, there’s also, there’s also then the patients that come in and they just, they’ve very medicalised their problem. And, they think the physio is the person that needs to fix it. And, as much as you try and come in with some open questions, and things, they just, they wanna shut it right down and focus it back towards what the physio or the, the Healthcare can do for them [Staff interview 7]. |
|  | Patient | Wasn’t a beneficial pathway for me, and that’s what I said. I said, “I, I need, I need somebody face to face,” and, as I said at the time, it’s like somebody telling you to do Yoga, because it’s gonna be marvellous for you, but doesn’t explain to you what it is. Um, and I think, I think, you know, as I say, I think this, this lack of interaction, this lack of face to face [Patient interview 2]. |
|  |  | I’m not sure if that’s a perfect fit for everybody, it certainly wasn’t for me. Because I needed, I needed to talk through what I was doing, I needed to find out if I was doing, what I was doing, was right. Um, because with myself, because of my spinal injury, it led on to me having um, additional problems, with my mobility and my legs. Um, so, I was a bit nervous, if you like, of some of the exercises, cos I didn’t know whether I was doing it right or wrong. I need somebody to tell me, I need a professional, to hold my hand really, in the beginning [Patient interview 6]. |
| **Theme 5**  **Patient autonomy** | Physiotherapist | Taking ownership and responsibility for their own health, rather than being told, I think, so that’s only a good thing. ‘Cause, it’s got to be them that want to change and do stuff, hasn’t it, rather than me. I think if they’re choosing what they want to do and they’re taking ownership of it, and it’s something that’s going to help them long term, that’s going to be a benefit. [Staff interview 2]. |
|  |  | I think again, it comes down to for a certain sector of the patients that we see, it would suit certain people very well. And it gives them a choice and it gives them a choice of another erm, choice of a way of improving their health status. Erm so it’s, yeah it’s having an extra tool in the box isn’t it? To help people go in the right direction in terms of their health. So, definitely it’s got a place. Absolutely. Because the, at the moment, the digital programmes that we’re using is, we decide, you know [Staff interview 8]. |
|  |  | There’s also then the patients that come in and they just, they’ve very medicalised their problem. And, they think the physio is the person that needs to fix it. And, as much as you try and come in with some open questions, and things, they just, they wanna shut it right down and focus it back towards what the physio or the, the Healthcare can do for them [Staff interview 7]. |
|  | Patient | By using, or being able to access the MyJointPain website, going forward, I’m able to manage my health much better. Whereas, I think if I’d just gone down a straight physio route, I wouldn’t be where I am now, you know, I’m, I’m confident about my future and, and being able to make decisions for myself about things, never the proviso, I would go to a Health Professional, if I was concerned. But I just feel that it’s given me self-management, and I’m, I’m someone who likes to be able to manage things myself [Patient interview 1]. |
|  |  | I’m just, you know, trying and, and if, if they’re really painful I won’t do them. So that’s what I mean, that maybe, maybe if a physiotherapist was saying yes, it might be painful now but if you like pursue it might easier or, you know. But that’s what I mean, that I just feel like maybe too much responsibility is on my shoulders on choosing them [Patient interview 7] |
| **Theme 6**  **Future use of TRAK MSK** | Physiotherapist | I think once patients have been seen once or twice face to face, I definitely think it’s easier then to introduce virtual, so I wonder whether a combination, like an initial appointment where we can show the patients the website, get them used to navigating it and then following them up virtually. I think a balance would, would, erm, probably encompass, erm, and help to meet the expectations of more patients. I think some patients would be very happy to be seen virtual, but I think using a combination would help to, erm, capture more patients [Staff interview 9]. |
|  |  | It’s the choice, to swing between you know, you may ha, you may have a couple of appointments with somebody face-to-face and actually, you’ve built the confidence around them being able to then carry on their contact digi, you know. Using digital, you know, the digital programme and Bridges, you know. So, you may start off them coming here, traditional physio and then end up using this further down the line once they’ve built their confidence etc. [Staff interview 8]. |
|  | Patient | I mean, there’s a mass of stuff there, but it’s picking the right ones, and then having somebody to talk to, if you’re not entirely sure, that, oh, that you are doing the right thing. I think a combination would be better, [Patient interview 8] |
